# "Slopes": a new approach to scoliosis radiographic measurement and evaluation, related to the horizontal plane in a bodily view

**DOI:** 10.1186/1748-7161-8-S1-O29

**Published:** 2013-06-03

**Authors:** S Negrini, S Atanasio, S Donzelli, F Zaina

**Affiliations:** 1 University of Brescia, Brescia, Italy; 2 IRCCS Don Gnocchi, Milan, Italy; 3 ISICO (Italian Scientific Spine Institute), Milan, Italy

## Background

Recently, the sagittal evaluation of the spine moved from a regional spinal view (curves) to the bodily view (“sagittal balance”), looking at the relationships between the gravity force (vertical or horizontal lines) and pelvic, and spinal, parameters. To move the analysis of the frontal plane from a spinal to a bodily view, we introduce the concept of “slopes”: inclination of the spine with respect to the vertical line (used in the past to obtain the measurement of curves with the Ferguson Method); we measured it using the End-Vertebrae Angle (EVA - i.e. the inclination of the end scoliosis vertebrae with respect to the horizontal line).

## Aim

To check the relationships between slopes and Cobb degrees measurements and SRS-Ponseti classification.

## Methods

404 scoliosis frontal radiographs, randomly chosen out of a database of 1,008 scoliosis patients under 18 years of age, were measured. Due to low quality image, 6 were excluded. Curves ranged 5°-66° Cobb. The T1, S1 and all limiting vertebrae slopes were measured. We also considered the difference of two slopes included in the same scoliosis curve (SCD), their location, and the number of main slopes. Slopes were considered secondary if they were 5° or more inferior to another one.

## Results

We found differences between the slopes in the same scoliosis curve at thoracic (48% proximal, 52% distal, P<0.01) and thoraco-lumbar (44%-56% respectively; p<0.0005) levels, with a tendency at proximal thoracic (46%-54%). SCD ranged 0-19°, with an average of 4.3°: it increased in caudo-cranial direction (4.1° lumbar L; 4.9° proximal thoracic PT). From 44% (L) to 55% (TP) slopes had a 5° or more difference in the same curve, and 10 to 14% had 10° or more. Slopes were located mainly in T11 (18.3%), L4 (11.9%), L3 (11.8%), and T12 (11.0%). We had 42.3% single, 56.9% double, and 0.8% triple curves, while primary slopes were 19.6%, 44.1%, and 34.8 respectively, with 1.5% quadruple.

## Conclusions

Slopes are not symmetric in scoliosis curves. The difference existing between the two slopes of the same scoliotic curve can have therapeutic, but also prognostic and etiologic implications.
